# Bring Your Non-self to Work? The Interaction Between Self-decentralization and Moral Reasoning

**DOI:** 10.1007/s10551-021-04975-1

**Published:** 2021-10-30

**Authors:** Mai Chi Vu, Nicholas Burton

**Affiliations:** Newcastle Business School, Northumbria University, Newcastle-upon-Tyne, NE1 8ST UK

**Keywords:** Non-self, Emptiness, Buddhism, Moral reasoning

## Abstract

Spirituality continues to exert a strong influence in people’s lives both in work and beyond. However, given that spirituality is often non-formalized and personal, we continue to know little about how moral reasoning is strategized. In this paper, we examine how Buddhist leader-practitioners interpret and operationalize a process of self-decentralization based upon Buddhist emptiness theory as a form of moral reasoning. We find that Buddhist leader-practitioners share a common understanding of a self-decentralized identity and operationalize self-decentralization through two practices in Buddhist philosophy—skillful means and the middle way—to foreground social outcomes. However, we also find that practitioners face tensions and challenges in moral reasoning relates to agency—the ‘re-centering’ of the self as an enlightened self and the use of karmic reasoning to justify (un)ethical behavior—and contextual constraints that lead to feelings of vulnerability and exclusion. We present a model that elaborates these processes and invite further research that examines novel approaches and dynamic interpretations of the self in moral reasoning.

## Introduction

An individual’s spirituality provides an ontological basis for a sense of self which can influence an individuals’ position in society and their attitudes and behaviors in organizations (Alshehri et al., 2020). Indeed, spirituality is a dynamic process of meaning-making through a complex negotiation between one’s self and moral values, and through interactions with others and the wider social system (Karakas & Sarigollu, 2019). The idea of the moral self-concept also reflects both the subject and the object of moral decisions (Rozuel & Kakabadse, 2010; Vice, 2003) and reflects a crucial component of moral identity (Fernandez-Duque & Schwartz, 2016a, 2016b) which influences an individuals’ process of moral reasoning (Schwartz, 2016). However, business ethics research has paid relatively little attention to the importance of self in moral reasoning, with a few notable exceptions (e.g., Gozdz & Frager, 2003; Noval & Hernandez, 2019; Rozuel & Kakabades, 2010; Sekerka & Bagozzi, 2007), and moreover, there are limited studies exploring the mechanisms and interactions between spirituality, self and moral reasoning (e.g., Alshehri et al., 2020; Parboteeah et al., 2008; Steffy, 2013).

Beyond this surprising gap in the business ethics literature, the spirituality at work (SAW) literature has often taken a narrow perspective towards the link between self and spirituality, embracing ideas that spirituality enables an individual to realize an essential self (Bell & Taylor, 2003), journey on a process of self-discovery (King & Nicol, 1999; Neal, 1997), and develop self-insight (Zellers & Perrewe, 2003) or self-knowledge (Dehler & Welsh, 2003). Thus, the continued focus on an all-too-often idealized and uncritical interpretation of a spiritual self-identity is partial at best. Further, while it is often argued that spirituality is a key component of ethical behavior (Gull & Doh, 2004; Furnham, 1996; Zsolnai, 2011) as it relates to personal morality (Giacalone & Jurkiewicz, 2003), value judgements (Wood & Bandura, 1989), and empathy with others (Otaye-Ebede et al., 2020), spirituality can also lead to (im)moral or (un)ethical action (Vu, 2021a; Zhang, 2020). It is also possible that when there is an overemphasis on the self, it is more likely that the ego can take over the focus of an individuals’ consciousness so that moral decisions may no longer be genuine and instead respond to personal interests (Rozuel & Kakabadse, 2010).

Given these surprising gaps and uncritical treatments, we utilize the Buddhist theory of emptiness as an analytical tool to explore how Buddhist leader-practitioners in organizational contexts decentralize the self in constructing and negotiating self-identity to avoid being trapped in a sense of ego that may blur moral reasoning. Unlike the dominant assumptions of self-idealization that are prevalent in the SAW literature, Buddhist notions of self-decentralization reject the very idea of self through the way in which suffering arises from the illusion of a definitive self (Vu, 2021b). Emptiness theory claims that all phenomena, including self-hood, are empty of intrinsic existence (Van Gordon et al., 2016). *Self-decentralization* has also been under-explored in the literature (Alvesson & Willmott, 2002; Brown, 2019; Vu, 2021b), and we were motivated to unpack the question of how Buddhist leader-practitioners make sense of self-decentralization and how it shapes moral reasoning. While there has been a huge increase in mindfulness scholarship in management and organizational studies (e.g., Dane & Brummel, 2013; Kudesia, 2019; Vu & Burton, 2020), Buddhist philosophy encompasses a much broader range of spiritual practices that should equally interest management scholars.

We undertook a qualitative study of 53 Buddhist leader-practitioners in Vietnam across a range of organizations and industry sectors. To signpost our contributions, our findings show that Buddhist leader-practitioners share a common interpretation of self-decentralization informed by the Buddhist practices of *Skillful Means* and the *Middle Way* which influences their strategies of moral reasoning and help them respond to heterogeneous tensions in the workplace. Thus, we argue that self-decentralization is a form of moral reasoning enacted through the practices of *Skillful Means* and the *Middle Way*, and which results in moral reasoning that foregrounds social outcomes. However, we also find that the work organization creates significant contextual tensions such as perceived social exclusion which lead to pressures to ‘re-center’ the self as ‘enlightened’. These contextual pressures lead to changes in moral reasoning from the collective to self-centred concerns. We develop a theoretical model of self-decentralization as a moral reasoning strategy that captures these dynamics in action.

Our paper proceeds as follows: we begin by reviewing the literature on self and moral reasoning. We follow this with an introduction to the Buddhist emptiness theory that can extend our understanding of the notion of the self. Next, we outline our context and elaborate our research method. We then offer an extended discussion, and conclude with pathways for future research.

### Self, Agency and Context

The concept of self is a dynamic system that involves affective and cognitive structures that consist of and organize self-identity (Baumeister et al., 1998; Gecas, 1982). Individuals develop a self-concept of who they are—to themselves and to others—that embodies a set of meanings, inferences about who we are, how others act towards us, our wishes, desires, and idealized views of who we are (Leary & Tangney, 2011; Stets & Burke, 2014). Individuals often construct and sustain self-identities that are congruent with their self-concept (Snow & Anderson, 1987), striving for a unique and stable identity (Brown, 2019). As self-identity often involves gender, ethnicity, social position or religious/spiritual affiliation (Baumeister, 1986), interpretations of self-identity reflect both culture and context.

Since the self is emphasized in identity construction, individual agency has become an important aspect of how self-identity is constructed as it relates to how individuals generate an ongoing sense of the self through interactions in social contexts (Brown, 2017, 2019; Ybema et al., 2009) and make behavioural choices (Leary & Tangney, 2011). Within this agentic interpretation of self, individuals engage in creating, presenting, and sustaining a personal identity as they attempt to construct a definable self (Driver, 2013; Knights & Clarke, 2014), and negotiate conflicting and coexisting elements that can lead to ego-defence, fantasy and self-projection (Petriglieri & Stein, 2012). For example, in organizational contexts, individuals can involve themselves in identity games to preserve and protect the self by constructing their self-identities in response to the heterogeneity of networks, relationships, and organizational expectations (Gergen, 1991). Leaders, for example, may fantasize about self-aggrandizement (Brown, 1997) or grandeur (Maccoby, 2000). These kinds of narcissistic self-conceptions (Steyrer, 2002) may trigger an ego-centric or idealized portrayal (Schwarz, 1990) demonstrating self-idealization. In other words, individual agency is strongly embedded in how individuals manufacture, regulate, produce, design and enterprise their selves (Alvesson & Wilmott, 2002; Brown, 2017, 2019).

In the work organization context, a self-idealized identity is also triggered by social interactions that challenge individuals’ enactment of their identities (Bartel et al., 2012; Ollier-Malaterre et al., 2013), reflecting how self-identity is constructed in contextual negotiation (Karreman & Alvesson, 2001). For example, individuals respond to institutional pressures (Voronov & Vince, 2012) and are conditioned by social and power relations and professional experiences (Bourdieu, 2000). Individuals tend to selectively modify and accommodate multiple and fragmented self-identities in a continuous dialectic of context and agency, such as improvising or crafting self-identity to a set of customized and individuated selves (Brown, 2015). As a result, individuals are trapped in an ongoing quest for a self-centered identity that assumes the self is existentially significant (Alvesson & Robertson, 2016). Given that self-identity is “imperilled, menaced and fragile” (Brown & Coupland, 2015, p. 1316) and often constructed to align with organizational ends (Butler, 2009) and negotiated in the context of issues of control and resistance (Brown, 2019), there are many selves embedded in individuals that represent different aspects of the prevailing social structure (Stryker, 1980). Self-identity is also fragmented because it is fluid, dynamic, context-dependent, interpreted reflexively (Brown, 2019; Giddens, 1991; Karreman & Alvesson, 2001), and regulated by individuals’ feelings, beliefs and behaviours (Liu & Robertson, 2011). As Keenoy et al., (2009, p. 395) noted, the identity construction process is reflexive, complex, multifaceted, transient, creating and re-creating a process of identity-construction that is “negotiated and re-negotiated in the dynamic interplay between internal strivings and external prescriptions, between self-presentation and labelling by others, between achievement and ascription, and between regulation and resistance”.

### Self and Moral Reasoning

The notion of the self and how individuals regulate the self are closely associated with moral behavior (e.g., Aquino et al., 2009; DeTienne et al., 2019; Floyd et al., 2013; Joosten et al., 2014)—taking action that is perceived as ‘right’ (Craft, 2013; Crossan et al., 2013). Morality is often considered as universal and obligatory (Ash & Woodward, 1987; Piaget, 1977), and includes perceptions about justice and welfare, and beliefs and behaviours related to treating others fairly and respecting their rights (Turiel, 1983). Studies show that individuals who place moral values and standards central to their self-concept tend to display more prosocial behaviors than those who do not (e.g., Aquino et al., 2009; Jordan et al., 2011). For instance, if an individual acts consistently with his or her moral values (e.g., benevolence, honesty, forgiving, and helpful), rather than pursuing self-interest values (e.g., achievement, being successful and ambitious), he or she possesses a moral personality (Blasi, 2009).

The notion of self can be considered as a mediator of moral reasoning and action (Blasi & Glodis, 1995) because individuals often feel morally responsible based on their moral beliefs (Hardy & Carlo, 2005). Based on individuals’ moral beliefs (e.g., spiritual/religious orientations), they can express a sense of humanity by being morally responsible for avoiding harming others (Small & Lew, 2021). Connectedness to the self also influences moral capability (Terestchenko, 2008). For instance, in the absence of connecting to the self, individuals can compartmentalize their ability to exercise moral judgment and moral behavior. While connectedness to the self is considered a necessary condition for authentic moral practice, connectedness to the self in a sense that the ego takes over the focus of consciousness often results in individualistic moral decisions, or decisions that conform to collective expectations (Rozuel & Kakabadse, 2010) and thus risks personal moral integrity (Reed et al., 2007; Treviño et al., 2006).

Based on a rationalist view, ethical decision-making is a process of moral reasoning and moral reasoning is one of the strongest predictors of ethical behavior (Shao et al., 2008). The literature on moral reasoning is familiar with moral rationalization—condoning wrongdoing (Bandura et al., 1996) and moral decoupling—dissociating judgments of morality from judgments of performance (Bhattacharjee et al., 2013)—to alleviate dissonance triggered by immoral behavior (Lee & Kwak, 2016). Studies also show that people can act in ways that are harmful to others in situations where such behavior can be rationalized (e.g., Batson et al., 1997; Detert et al., 2008) since moral behavior can be determined by the situation and context (Doris, 1998; Harman, 2003) and individual characteristics (e.g., Walker & Frimer, 2007). Such strategies raise tensions and dissonance when individuals have to compromise their moral standards (Burton & Vu, 2020; Lee & Kwak, 2016). While tensions in moral reasoning have been addressed in the literature, such as between self-serving moral reasoning and authentic moral values in weighing different approaches, between a moral self-concept and rationalizing immoral behavior or compromising one’s moral standards (Cowan & Yazdanparast, 2019; Thøgersen, 2004), the extent to which internal tensions influence ethical evaluations (Cowan & Yazdanparast, 2019) and how different cultural contexts influence ethical decision-making (Vitell et al., 2016); it remains unclear how spirituality and the notion of the self from a spiritual perspective link to strategies of moral reasoning.

### The Self and Spirituality at Work

Spiritual practice embeds moral and ethical conceptualizations of self (Corner, 2009; Sheep, 2006) as it is one of the tools to affirm the self (Pace, 2013). Much of the SAW literature argues that spirituality reflects an inner source of energy (Giacalone & Jurkiewicz, 2003), to attain a sense of transcendence (Dehler & Welsh, 1994), wholeness (Neal, 1999), and fulfilment (Howard, 2002), which encourages an integrated or whole self (Brown, 2003; Lips-Wiersma, 2003). Further, the literature advances the idea of bringing a complete self to work (Kinjersky & Skrypnek, 2004; Neal, 1999) but this has been suggested to lead to legitimate concerns over how spirituality has become an instrument for organizational ends (Lips-Wiersma et al., 2009). Similarly, Driver (2005) argued that the premise that spirituality in organizations can reveal an authentic self is ‘empty speech’ (Driver, 2005). Driver argues that organizations often have instrumental ends in mind when they encourage the idea of a bringing a whole self to work (Dehler & Welsh, 1994; Delbecq, 1999) rather than one in which an individual can pursue larger purposes. In fact, in such circumstances the ego simply becomes attached to a larger identity known as the organization and its purposes (Driver, 2005), and each individual is fragmented by small egos which can result in adverse emotional states and repressed personalities (King & Nicol, 1999). This puts an individual in situations where compartmentalization may occur between an individuals’ moral values and collective expectations affecting the moral capacity of individuals (Schwartz, 2016).

Given the controversial discussions around the notion of the whole self in the work context and the limited examination of how spiritual interpretations of the notion of the self influence moral reasoning, we explore how Buddhist interpretations of the ‘self’ can potentially impact on individuals’ reasoning, We have chosen to examine practitioners from the Mahayana Buddhist tradition as Mahayana Buddhist conceptualizations of *non-self* and *emptiness* can provide an alternative view to existing dualistic interpretations of moral values leading to tensions in organizations. Such a view rejects the subject-object structure of the self and liberates the self from ego’s preoccupations with a non-conceptual insight to enable individuals to see things as they are in the situated, conditioned nature of propositional claims, rather than in a dualistic manner with over-attachment to fulfilling either individual or organizational pursuits.

### Emptiness and Self-decentralization—Buddhist Interpretations of Self-identity

Within Buddhism, there are a variety of philosophical schools (e.g., *Vaibhāṣika* and *Sautrāntika* from the Hinayana school; and *Madhyamika*^1^ and *Yogācāra*^2^ from the Mahayana school). In this paper, we focus on the philosophy of Madhyamika known as the Middle Way to unpack the notion of emptiness and its relation to the self-concept. The “Middle Way” (Madhyamika) school of philosophy propounded by Nāgārjuna is based on the insight of emptiness (*sunyata*), dependent arising (*pratitya- samutpada*) and nominal-verbal designation (*praj-napti*)^3^ (Garfield, 1995). Based on these insights, the Middle Way perspective highlights how a phenomenon is both conditioning and conditioned by others. This perspective is neither merely ontological nor epistemological, neither substantialist nor nihilistic, neither existent nor non-existent, and without absolute dualisms in the world of conditional relativity (Chinn, 2006; Garfield, 1995; Nagao, 1989). It is a relational-processual perspective. The Middle Way rejects both extremes of substantialism and nihilism, implying a balanced view and approach to life. However, it is important to point out that the Middle Way does not mean a compromise or a mid-point between extremes, but the approach is to overcome extremes by transcending the dualistic view towards the non-dual ultimate truth. The Middle Way is demonstrated in Nāgārjuna’s Mūlamadhyamakakārikā (verses on the Middle Way), verse 18 of Chapter XXV:

‘Whatever is dependently co-arisen, that is explained to be emptiness. That, being a dependent designation, is itself the middle way’ (p. 69, 93, 304).

Emptiness, as dependently co-arisen, is termed a nominal designation as it lacks essence, or independent existence, depending on verbal reference or conventional characterization for its existence (Garfield, 1995, p305). Nāgārjuna argues this view in Mūlamadhyamakakārikā, chapter XXII:

‘Empty should not be asserted. Nonempty should not be asserted. Neither both nor neither should be asserted. They are only used nominally.’

The Madhyamika school of Mahayana Buddhism propounded by Nāgārjuna is also known as the theory of emptiness (*Sunyavada*). Emptiness (Pāli: *suññatā,* Sanskrit: *śūnyatā*) is a fundamental Buddhist teaching that all phenomena, including the ‘self’ are “empty” of intrinsic existence (Thich, 1999). Emptiness is associated with all existence (Garfield, 1995); it is a state of being, a way of life and truth of existence (Van Gordon et al., 2016), and yet it is one of the most poorly elucidated and interpreted Buddhist concepts in the literature (Shonin et al., 2015). Following Nāgārjuna’s Mūlamadhyamakakārikā, emptiness is asserted to be dependent and nominal, conventionally existent but ultimately empty (Murti, 1973). Emptiness is also a skillful means (*upaya*) disclosing the non-substantiality of phenomena, freeing oneself from unsatisfactory attachment and clinging. The Middle Way can contribute to further understandings of moral relativism (Brogaard, 2008) such as the relative expressions of ‘right’ and ‘wrong’, the contextual variability of truth-values in moral judgments, and the apparent faultlessness of moral disagreement as there is no fact of the matter as to whether the utterance is simply true or false because all phenomena are empty in nature.

The Buddhist perception of ‘truth’ in the theory of emptiness consists of the search for *ultimate truth* (*paramattha*-*sacca*, -*vacana*, -*desanā*)—that ‘life is empty’ through the enlightened eye. It marks a departure from Western interpretations of how the material mind is ‘embodied in a brain dependent on material causes and conditions whereas Buddhism interprets the ultimate truth that the mind is ‘empty of specific materiality […] it is the source of all there is’ (Schuyler, 2012, p. 6). The *ultimate level of truth* in Buddhism is a vehicle for understanding Buddhist metaphysics and epistemology (Garfield, 1994). From a Buddhist perspective, mental representations of the self as a desirable object is incompatible with the impermanent nature of reality, and the avoidance or removal of such a mental fixation encourages a more objective perception, compassion, and a reduced selfishness that avoids or alleviates suffering (Sahdra et al., 2010).

Therefore, in Buddhism, individual ego or self-hood has no independent or inherent existence, but exists in relation to all others in a context of continuous change (Cooey, 1990). In Buddhism, there is a metaphysical position that denies the ontological reality of the self (Ho, 1995). Siderits (1997) presents two views of the Buddhist approach to the ‘self’: (1) Buddhism denies the existence of independent selves as reductionism because ‘wholes’ are nothing more than their atomic parts that are conventionally real but ultimately unreal; and (2) rather than being a metaphysical thesis that claims nothing has an independent existence on its own, the Buddhist emptiness theory is a semantic thesis reaffirming that no statement about ultimate reality is true. Buddhism rejects the assumption that personal identity entails a personal uniqueness in comparison with other individuals (Banaji & Prentice, 1994). Such an assumption is a contextual perception that is subject to change and empty in nature. Self-decentralization helps to deconstruct the self, the internal mental state of an individual (Vu, 2021b) and allows the individual to consider the external consequences of actions by embracing interconnectedness which is rooted in moral responsibility (Gould, 1995). Moral responsibility refers to a feeling of obligation (Williams & Gantt, 2012), the consideration of actions and decisions in relation to personal causality (Brees & Martinko, 2015) and the need to consider others (Fasoli, 2017a, 2017b). Studies show that Buddhist practices (e.g., mindfulness) can predict an individuals’ moral responsibility (e.g., Small & Lew, 2021), however, no studies have examined the influence of the practice of non-self on moral responsibility even though moral responsibility is an important component of the self-concept (Fernandez-Duque & Schwartz, 2016a, 2016b). By letting go of a sense of the need to have a self or an ego, non-self extends moral responsibility beyond individual motives (Fasoli, 2017a, 2017b), to be responsible for other individuals without individual greed, aversion or delusions of the existence of a ‘self’ (Purser & Milillo, 2015).

## Methodology

To explore ideas of self-decentralization and its impact on moral reasoning, we collected interview data from individuals who described themselves as having a leadership or executive role of an organization in Vietnam.

### Research context

We selected Vietnam because of the rising ‘Engaged Buddhist’ movement in the country that facilitates Buddhist practices in organizations (Vu, 2021a, 2021b; Vu & Tran, 2021). Vietnamese Buddhism is often associated with Thích Nhất Hạnh’s introduction of Buddhist practice and Zen Buddhism. However, it is important to note that Thích Nhất Hạnh’s movement represents a more elite, cosmopolitan strain of Vietnamese Buddhism that is quite distinct from the way Buddhism is understood and practiced by most Vietnamese (Soucy, 2017). Vietnamese Buddhism has an ‘ethnic’ nature and religious pluralism as it blends with a mix of Confucianism, Taoism, Vietnamese animistic beliefs (He et al., 2011), and folklore religion (e.g., worshipping of ancestors, goddesses, local deities, spirit-possession, etc.) (Cleary, 1991; Kendall, 2011). While there are several schools of thoughts like Pure Land (*Tịnh Độ Tông*) of the Mahayana school and Tantra of the Vajrayana school (Cleary, 1991), the core teachings and practice of Buddhism in Vietnam revolve around core concepts such as the Four Noble Truths, the Noble Eightfold Path, and karma.

While Vietnamese Buddhism is known to be embedded in folklore traditions and the Chinese concept of ‘the three religions with the same root’ (*tam giáo đồng nguyên*) during the Lý-Trần dynasties (1009–1400) (Nguyen et al., 2013), in the contemporary context, Buddhist practice has become more influential due to its emphasis on practicality and flexibility (e.g., Vu & Tran, 2021). On the other hand, Taoism with its emphasis on avoiding conflict, Confucianism with feudal (*phong kiến*), corrupted and misguided characteristics under communism, and the prevalence of folk superstitions have been considered as causing backwardness (Leshkowich, 2006) and have become less influential in the Vietnamese society (Nguyen et al., 2013; Yong-zhang, 2002). In this study, we focused on participants from the Mahayana tradition as the engaged Buddhist movement in Vietnam (Vu & Tran, 2021) reflects the application of Buddhist principles and philosophies that attend to contemporary sufferings due to social, political, economic or environmental issues (Thich, 1999) and the Mahayana path facilitates not only individual progression but also through doing good for others through skillful and contextual applications that are perceived as more practical in complex business contexts.

After the ‘Đổi Mới’ policy in 1986 Vietnam has been a transitional economy, moving from a state-controlled to less restricted market-orientation. However, unlike its economic reforms, reforms to the legal and regulatory systems have not generated well-functioning markets (Trubek & Santos, 2006), leading to ineffective law enforcement resulting in institutionalized corruption (Cuadra et al., 2010; Vu, 2021a) and a reduced level of trust in institutions and society (Vu & Tran, 2021; Vu, 2020). Within this context, self-identity construction and the practice of non-self is inevitably surrounded by tensions around moral responsibility and moral actions (Chu & Vu, 2021), which is worth examining. We recruited fifty-three participants from across twenty-eight organizations across a number of different industries. The profile of our interviewees is shown in Table 1.

**Table 1:** Respondents’ information

Person	Gender	Position	Company	Sector
B1	M	CEO	VN1	Construction
B2	M	CEO	VN2	Pharmaceutical
B3	F	Marketing Manager		
B4	F	Public Relations Lead		
B5	F	CEO	VN3	Education
B6	M	Financial Manager	VN4	Hospitality
B7	M	Director	VN5	Advertisement
B8	F	Deputy Director		
B9	M	Managing Director	VN6	Management Consultancy
B10	F	Branch Manager	VN7	Pharmaceutical
B11	F	CEO	VN8	Law
B12	M	Corporate Case Manager		
B13	M	General Director		
B14	F	Country Manager	VN9	Manufacturing
B15	M	Production Manager		
B16	F	Relationships Manager	VN10	Banking and Finance
B17	M	Chief Accountant	VN11	Transportation
B18	F	Manging Director		
B19	M	Financial Manager		
B20	F	CEO	VN12	Financial Services
B21	F	CEO	VN13	Publishing
B22	M	Deputy Director		
B23	F	Human Resource Manager		
B24	M	Director	VN14	Hospitality
B25	F	Human Resource Manager		
B26	M	Customer Service Manager		
B27	M	Marketing Consultant		
B28	M	Production Manager	VN15	IT Consultancy
B29	M	Managing Director	VN16	Education
B30	F	Marketing Manager		
B31	F	CEO	VN17	Construction
B32	M	Design Manager		
B34	F	Branch Manager	VN18	Pharmaceutical
B35	M	Expert Consultant	VN19	Banking and Finance
B36	M	Corporate Finance Senior Advisor		
B37	M	VIP Customer Relations Manager		
B38	F	Credit Manager		
B39	F	Branch Manager	VN20	Management Consultancy
B40	F	Project Manager		
B41	M	Credit Manager	VN21	Financial Service
B42	M	CEO	VN22	Manufacturing
B43	F	Managing Director	VN23	Hospitality
B44	M	Deputy Head of Marketing		
B45	F	Head of Customer Service		
B46	F	Head of Relationship Management		
B47	M	Product Manager	VN24	Banking and Finance
B48	M	Project Manager		
B49	M	Head of Training of Trainers	VN25	Education
B50	M	Product Manager	VN26	Pharmaceutical
B51	F	Manager	VN27	Retail
B52	M	CEO	VN28	Construction
B53	F	Financial Manager		

### Sample

The participants of our study were all Buddhist practitioners who practiced Mahayana Buddhism^4^ for more than twelve years. The Mahayana school guides practitioner’s through direct experiences instead of an abstract ideal in discovering the unconditional worth of persons (Vokey, 2001). Participants referred to the Sūtras from the Mahayana tradition (e.g., the Heart Sūtra—Prajñāpāramitāhṛdaya (*Bát Nhã Tâm Kinh*); the Lotus Sūtra—Saddharma Puṇḍarīka Sūtra (*Diệu pháp Liên hoa kinh*); the Flower Garland Sūtra—Avataṃsaka Sūtra (*Kinh Hoa Nghiêm*); and the Laṅkāvatāra Sūtra (*Kinh Lăng Già*))—to unpack the process of self-decentralization through the practice of non-self. The Mahayana metaethics embraces the idea of ‘seeing things as they are’, appreciating the conditioned nature of propositional truth and the experiential knowledge of intrinsic goodness (Vokey, 2001). The Buddhist participants were recruited by snowballing between 2018 and 2019. The first twelve participants were recruited in a Buddhist business community group in Hanoi in 2018, and participants were asked to recommend further participants. Semi-structured interviews were utilized, and each interview was conducted in a private meeting room for about one hour at the participants’ place of work. We began the semi-structured interviews by describing to participants that we were interested in what the concept of self-identity meant to them, therefore, while we located our interview broadly within the field of ‘identity’, we allowed any connection between Buddhist practice and self-identity or moral reasoning to emerge spontaneously during the interview process. In other words, we avoided imposing concepts such as ‘non-self’ or ‘emptiness’ on the interview as a priori ideas or explanations. We approached the interviews without an extensive interview schedule, preferring to adopt a largely unstructured approach. The initial question posed “What does the idea of self-identity mean to you?’ was consistent across the interviews, however follow-up questions varied in each interview in order to more deeply explore responses that related to our research interests. The interviews were conducted and transcribed verbatim in Vietnamese by the lead author and double-translated in English by the lead author and a translation agency. Differences in translation were resolved through discussion between the lead author and the agency.

### Data Analysis

Template analysis was used to analyze the transcribed interview data. Our coding followed the approach developed by King (1998, 2004) which has gained traction in multiple disciplines including management and organization studies (e.g., Burton & Galvin, 2018). Template analysis is a flexible type of thematic analysis that emphasizes hierarchal coding but balances structure with flexibility to adapt it to the needs of a particular research study. Preferring to allow themes to emerge from the data, we avoided pre-defining a priori codes. We created a template that subsequently enabled us to examine similarities and differences in narratives. The template was continually modified during the analysis phase. Where new themes emerged or other changes to the templates were made, previously analyzed interview transcripts were re-examined, and this iterative process continued *ad-finetum*. Each interview transcript was coded separately one at a time by each of the two co-authors, and differences in coding were resolved through inter-coder dialogue and discussion. We chose to code the data ourselves because we recognize that coding can sometimes be reductive, and we wished to stay immersed in the stories and narratives of participants in order to enhance the richness of the descriptions we produced. As an interpretivist and inductive study, we were primarily concerned with the richness of the narratives of participants and we judged that the epistemological flexibility of template analysis would allow us to balance a search for ‘integrative’ themes that permeated the data but at the same time not lose sight of interesting and unusual detail (King & Brookes, 2016). To support this aim, and given the complexities involved in both Buddhist philosophy and translation, we emailed a copy of the interview transcript to each participant to check for accuracy as well as examples of our coding (King, 2004).

Our final template is shown in Table 2.

**Table 2:** Coding template and themes

Integrative theme	Main themes	Example verbatim quotation
Interpretation of non-self	*Reduced ego-serving expectations*	Reducing my expectations is part of non-self because it helps me to empty out my ego and my personal expectations that come with it Expectations are suffering. The moment you cling to your expectations, you are attached to your ego. You suffer if your expectations are not met
	*Reduced ego-centric desires*	Non-self helps me out of my illusions. It constantly reminds me of my desires and my personal pursuits that can affect my life and my relationships at work The practice of non-self is aimed at eliminating suffering. Because you get rid of your greediness and your self-serving pursuits, you don’t have to wait for expectations to be met
	*Reduced ego-serving knowledge*	Ego is part of suffering. It controls your mental awareness and behaviour which can lead to harmful relationships at the workplace Non-self helps me to correct myself, my judgements and misunderstandings at my work
Agency Tensions	*Justifications*	I think about motives of my actions. It is crucial to identity whether I am serving myself or the benefit of others. What is the final outcome? Having a positive karmic consequence to benefit others is important As long as it generates good karma, the practice is working for me. The outcome is what proves your effort
	*Attachments*	Impermanence is the key to practice non-self. It is important to follow it and accept that there are external factors beyond control I cannot control everything. This is how I learn to practice non-self. My employees should learn to not always rely on me as well
Organisational Institutional Tensions	*Organizational constraints*	Non-self is practiced in caution and in a calculative manner, at least in my case because I have been taken advantage of so many times. You cannot compassionate all the time Non-self can be an easy target for others as well. If people do not understand why you are sacrificing things, especially other shareholders, they just exclude you gently step by step from important meetings
	*Institutional constraints*	Have to bribe to make something meaningful happen because this is how business works here The law enforcement is weak, this is why you can get away with bribery. What can I do? Sometimes you just have to go with the way people are doing business otherwise you are risking your employees’ job
Mechanisms	*Skillful means*	Wisdom and skilfulness is needed to master non-self. It is not a universal practice that you can apply as you see fit It is very important to know when you need to let go of non-self as well, especially if it becomes really personal that can frustrate others
	*Middle way*	Balanced expectations are crucial for non-self so that I am not overly attached to my ego but at the same time I am not practicing non-self at any cost No expectations are totally harmful or useful because it just means that I am letting go of my own effort as well. And that is not the essence of being a Buddhist practitioner

## Findings

### Non-self: Interpretation of Self-decentralization

When we asked participants questions about self-identity construction and organization life, they shared an interpretation of self-identity based on the notion of non-self. For example,

I do not have an identity because, based on the practice of non-self, having a self is just an imagination. (B4/VN2)

Participants understanding of non-self is shaped by the Heart Sūtra that helped them to identify the emptiness nature of the notion of the self’ at the ultimate level of truth rather than at the conventional level.

Non-self indicates that there is no self and no identity. […] the Heart Sūtra (*Bát Nhã Tâm Kinh*) guides me to identify the self as empty at the ultimate level of truth, it is empty of an essence of its own. […]. (B22/VN13)

In directly describing the ultimate truth in the Heart Sūtra, many participants relied on this doctrine to help them unpack the notion of the self: ‘Form is emptiness, emptiness is form’.

Participants explained that the idea of non-self shaped their interpretation of self-identity construction and encouraged them to recognize and *let go of ego-centric desires.* For example, a number of participants elaborated letting go of ego-centric desires in the following ways:

Non-self is a practice that helps to eliminate suffering. My suffering always comes from me asking myself ‘Why do I have to do this? Is it for my benefit or others?’ […] Realizing that I am not the centre of the universe changes the way I see things […] it makes me reveal my ego and the desires that manipulate my thinking and doing (B6/VN4)

I realized that once I am not obsessed with a ‘self’, I suffer less. I am less concerned about having to do certain things and especially trying to satisfy my ‘self’ and my ego. (B7/VN5)

These interpretations indicate a ‘right’ understanding of the practice of non-self and the ability to let go of ego and desires that ultimately cause human suffering. The participants also indicated that non-self is particularly important for their approach to leadership because it *rejects ego-serving knowledge*, which they conceptualized as a further form of suffering.

Practicing non-self is one way of how I correct myself, my judgments and my misunderstandings. This is particularly important in how I lead collectively with my employees because nothing works when I just follow what I think is right. In fact, everyone suffers from that […] questioning my ‘self’ and my practice helps me to recognize and experience emptiness, just like how in the Laṅkāvatāra Sūtra (*Kinh Lăng Già*), the Buddha taught Mahāmati about the emptiness of verbal interpretations […] (B20/VN12)

No one can be right all the time. Non-self teaches me exactly that. No good leadership can be sustained in isolation. Thinking you are right all the time is definitely a cause of suffering […] in fact being a leader is also empty based on the Laṅkāvatāra Sūtra (*Kinh Lăng Già*) (B42/VN22)

The above examples reflect how the concept of non-self rejects ego-serving knowledge and resists the idea of subordinate (and follower) relationships in the work organization context and how the Laṅkāvatāra Sūtra helps participants to critically question themselves, their approaches and practices to experience the state of emptiness. Many participants demonstrated an understanding of how the practice of non-self interacts with their leadership. Participants also emphasized how understanding the sources of suffering helped them to *reduce ego-serving expectations*.

Non-self for me is very simple. It is about reducing my expectations. No expectations, no suffering. (B1/VN1)

Before practicing non-self, it was all about myself, about how I can prove that I am different from other previous leaders of the company who were scared of taking risks. I wanted to be viewed as young and talented. But then there were too much pressures and suffering, which I realized was unnecessary. (B14/VN9)

As a manager I always have had high expectations of how I portray myself so that people look up to me […] When I expect a lot, I start worrying about when and how those expectations will be met and then I start to keep a close eye on my employees, which I believe sometimes had led to my unreasonable interference to push things forward or to speed up things. In all of those scenarios, I felt I always had something on my shoulder and I suffered from that burden. But with non-self, I considered my expectations as suffering and once I let go of them, it has been a relief. (B49/VN25)

By reducing ego-serving expectations, participants expressed the desire to move away from having to establish a leadership brand and to customize their personas to enhance motivation and confidence among stakeholders (Shamir et al., 1993; Sinclair, 2011). Participants’ responses reflect a Buddhist interpretation of expectations that are context-dependant. To illustrate, one participant remarked that:

Realizing that the self does not exist helps me out of my illusions. After all, Buddhist practice is about understanding the truths of the universe and non-self is one of those truths. (B31/VN17)

Participants’ expressions of self-decentralization demonstrates that a definite self is an illusion, and participants are aware of the importance of, and need to, decentralize the self and its associated ego-centric forms of suffering. Even though our participants shared an interpretation of self-decentralization, the operationalization of non-self is surrounded by tensions and challenges.

Non-self for me is a difficult practice. I see it as a life-changing Buddhist practice because it starts with me changing my own habits. […] You know it always come to the fact that everything we do normally is for that ‘self’. (B18/VN11)

To further explore these tensions, we begin with agentic challenges, and then proceed to highlight how contextual challenges to do with the work organization and institutional context impact the ability of our participants to fully exercise self-decentralization.

### Tension of Agency in Self-decentralization

Our findings highlight that individual agency deeply contributes to the formation of tensions due to the challenges of an ‘other-orientation’ and an over-attachment to Buddhist practice that can lead to the pursuit of an ‘enlightened self’ as a form of leadership branding.

#### Other-Justification

Findings show that tensions arise because participants rely upon *karmic consequences* as a *justification* for *making moral and ethical choices* in how they operationalize self-decentralization. The doctrine of karma in Buddhism ascertains that one’s certain actions have specific consequences (*karmaphala*): good action (e.g., praiseworthy action) accrues karmic merit and bad actions (e.g., hatred, violence, etc.) leads to karmic demerit (Finnigan, 2020). There are three elements of karma that participants considered in their moral reasoning: (1) the intention; (2) the act, and, (3) the completion of the final states of the act.

My decision to practice non-self is based on the 3 things: (1) I think about the motives of my actions—is it for the team or is it for me to impress someone? (2) I look at karmic consequences—does my action generate positive or negative karma? (3) I look at the possible alternative actions and its relevance to karmic consequences. So basically, my decision to practice non-self is based on motives and karmic consequences. (B31/VN17)

The above participant emphasized how moral acts have consequences as mentioned in a stock passage in the Pali canon (M.iii.178–179), which highlights the connection between karma and intention. Participants also recognized the need to deconstruct the ‘intent’ in karmic reasoning.

Understanding karmic consequences is very useful to see whether I am actually practicing non-self or not. You know when people do good things, it does not necessarily mean that they are good. Positive karmic consequences can come from deliberate motives knowing that doing A can lead to positive B that will be appreciated by many people, which is self-serving at the end. Non-self is when you accept to do harm to your ‘self’ for the benefit of others, not for doing ‘good’ that serve your image of ‘self’. (B50/VN26)

The above quote shows participants’ awareness in how karma can be produced by not only good intentions but also by volitional action that can reflect a possible sin in one’s heart without the performance of a physical act (Keown, 1996). Here, intent can be instrumental and karmic consequences can be determined by dispositions and moral virtue of individuals with which one performs the action and not the actions per se that generate consequences (Reichenbach, 1990). Therefore, recognizing the existence of ‘ego’ in intent is crucial to deconstruct the practice of non-self.

In this example, karmic consequences are located in a negotiation between individual and collective social outcomes. The notion of karma is used to justify an action that has good or bad consequences for oneself or for others. Participants used karmic reasoning in a utilitarian way to justify decisions that benefitted others, rather than adopting a self-centred perspective. This approach reflects the Mahayana perspective of participants in performing good deeds that indirectly involves others and dedicating the karmic merit to the benefit of others, not for the ‘self’ (Clayton, 2013). This approach was evident across our data with participants from the Mahayana tradition:

The most important thing for me is to practice non-self with a motive that reflects positive karma for the community. Motive with positive karma is not comparable to positive karmic consequences for the community. (B11/VN8)

As long as it generates good karma for everyone, the practice is working. (B37/VN19)

I tend to judge the overall positive karmic outcome for the community against any negative karmic motives that I myself have to consider. (B42/VN22)

However, this type of collective karmic reasoning encountered significant challenges in the work organization context. To illustrate:

To lobby or to please officials and distributors for example, sometimes I do not totally agree, but you cannot change your stakeholders so you might just want to respond to them positively for the benefit of our company. (B50/VN26)

Business is a game. Occasionally, you have to consider your organizational needs by following ‘best practices’ to gain approval for projects before your own values as it benefits others, not yourself. (B52/VN28)

While participants accepted the sacrifice of their karmic merit to benefit the community as a way to decenter the self, they did struggle in the negotiation between organisational/collective expectations and individuals’ moral values. The reason why they hesitated because they recognized that the ‘intent’ was not for social benefits, but for organizational instrumental gains. Certain organizational desires were evident in the above ‘intents’ that make the dispositions or intentions did not reflect moral self-transformation in good karmic deeds (Keown, 1996).

#### Over-Attachment

Many participants tended to rely on the notion of impermanence to give priority to external prescriptions or collective/organisational expectations and conditions in shaping their self-identity construction.

For me, everything is impermanent so there are things which are beyond my control no matter how hard I try. (B22/VN13)

When impermanence is overemphasised, our participants highlighted tensions between themselves and others which jeopardized group harmony and organizational outcomes. For instance:

My employees do not understand the impermanent nature of our life. They put a lot of effort into a project and get disappointed when it is rejected. I refused to contribute at the beginning to that project because I knew nothing that we do will change the outcome. I did not bother to tell them because I know they would not understand […] some see it as a bad leadership practice […] (B31/VN17)

It is important to exercise impermanence to master non-self. Without understanding how things are constantly changing, I don’t think anybody can let go of their ‘self’. That means, sometimes I have to be reluctant to recognize employees’ needs, […] and be more flexible in seeing what is right or wrong as everything can change […] (B19/VN11)

On the other hand, by being overly-attached to Buddhist practice, participants gave primacy to a strategized version of leadership that, rather than decentring the self, re-centres an ‘enlightened’ version of self as a form of leadership branding. This arises because leaders are also vulnerable to others’ opinions of them (Collinson, 2006) and they are attached to others’ expectations in forming leadership identity. For example, some participants shared an experience of how the practice of non-self may actually activate self-centralization if it is not practiced reflexively and context-sensitively:

To practice non-self, you have to acknowledge your failures and mistakes but I cannot do it on every occasion. It is sometimes beyond me to see my mistakes. Sometimes I think that I am practicing non-self but it turns out that it all comes back to the self. I am too passionate about practicing non-self and sometimes it fights back. For example, expecting my colleagues to do the same thing that I consider right is totally self-oriented. (B10/VN7)

Therefore, there are tensions arising from a lack of wisdom and skilfulness in Buddhist practice that can lead to counterproductive and painful experiences:

Non-self is not for everybody. You have to have a certain level of Buddhist practice and have enough experience to fully master it. Practicing non-self only partly is worse than not practicing it all because you can end up returning to your ‘self’ very easily without realizing it as a suffering. (B15/VN9)

As these examples highlight, the pursuit of non-self can bring tensions and counterproductive results, challenging practitioners’ viewpoint on what is right and wrong as they see things in a relative manner. Due to dynamic agentic tensions and the complex nature of the working environment, the practice of non-self can be constructed, lost, switched or even modified. Without the ‘right’ interpretation and practice, non-self can become an instrument that encourages the re-emergence of a self-serving and ‘enlightenment’ self that can influence and challenge practitioners’ moral values.

### Tension of Context in Self-decentralization

The multidimensional and complex nature of self-decentralization in an organizational context is shared by all respondents we interviewed. Beyond individual agency, participants identified organizational and institutional tensions that also influenced the operationalization of self-decentralization.

#### Organizational Constraints

The pursuit of non-self and the rejection of the existence of the self is extremely challenging. For example, one participant noted how:

Sometimes I am hesitant about the practice of non-self. Maybe because I have not been able to fully master it but people do take advantage of your good will. I covered for someone too many times and she just keeps taking advantage of me. So, for me, non-self is still practiced with caution and in a calculative manner, which I know is not what Buddhist non-self is fully about. But it is just so much harder in practice. (B15/VN9)

There is a tension that is centred around the fear of being vulnerable when participants practice non-self in leadership positions. For example,

People may think that being a leader means you have power. Yes, I do, I have the power to make things easier for people and they take advantage of it. You give them more budget, they don’t appreciate it. You give them more time, they become lazy. Having a soft side is sometimes a weakness in leadership and that is why practicing non-self is difficult. (B20/VN12)

I am cautious about non-self because people often take it for granted when you are being compassionate to them. Sometimes, my colleagues just tease me for being unrealistic in being too nice these days. I just feel like I could have been wiser in practicing non-self. In theory, you know what it is, but in practice, it is a different story. (B43VN23)

These examples highlight a tendency for leaders to conceptualize vulnerability as a weakness rather than as a strength that should be embraced. Many participants also struggled with the experience of being excluded or not being taken seriously in decision-making within the work organization for practicing non-self and cultivating a decentralized self. For instance:

It is hard to explain myself in meetings with board of directors who are not Buddhist and do not understand my practice of non-self. I have to explain myself very heavily if I initiate an idea that costs money or has a long-run return rate […] compromises on my side is inevitable, which sometimes involves the need to bribe officials to get something done […] (B18/VN11)

The things I struggle with is having to explain myself with my board of directors who see no meaning rather that earning profit. Occasionally I also realize that they conduct meetings without me to initiate projects they don’t think that I can fit in. (B13/VN8)

#### Institutional Constraints

Many participants in our study had an uncomfortable experience connecting their spirituality to the transitional context of Vietnam. For example,

Practicing non-self is painful. I have to bribe sometimes to make something meaningful happen. (B5/VN3)

I feel like I have to compromise my own values to get product approval when I have to take part in meaningless lobbying activities. For me, it is part of non-self because I sacrifice my own values to support my organization, however, it does not always feel right. It is just impossible to make such sacrifices, especially when it does not bring any benefit to the community or customers rather than just bringing more profit for the firm. (B15/VN9)

In these examples, acting morally and ethically are continually negotiated in the institutional context of corruption, reflecting the paradox of attaining organizational outcomes in weak institutional contexts, while maintaining personal values. We now discuss the mechanisms that participants used to resist the organizational and institutional tensions they experienced.

### Mechanisms for Ultimate Decentralized Self-identity Construction

Our participants highlighted two mechanisms through which they tried to attain self-decentralization and resist the contextual constraints they faced.

#### Skilful Means

All participants we interviewed embraced the practice of skilful means in guiding them to find a skilful approach in resisting tensions. They explained skilful means as:

Skilful means is the best contextual mechanisms that Buddhism has to offer. The Buddha used skilful means to teach his followers differently. There is no one best way to practice self-decentralization. My practice of non-self has to be put into context. To give you an example, in some occasions, I bribe because it is reasonable and worth it when the approved projects can help thousands of people, but in other occasions when it has limited or no social benefit, I refuse to do so […] the way I practice non-self is negotiated against karmic merit of outcomes, not karmic merit of myself. […] (B21/VN13)

Skillful means that participants demonstrated in our findings reflects *upāya* in the doctrine of ‘skill-in-means’ (*upāya-kausalya)* in the Mahayana Buddhist literature, a technique used by the Buddha to respond to a variety of karmic differences of his followers (Schroeder, 2004) to benefit different individuals in different contextual situations. The practice emphasizes contextualization based on the understanding that Buddhist practice(s) (*the dharma*) is empty of any substance of independent validity and its applicability is subject to its rhetorical, pedagogical and soteriological context (Schroeder, 2004). Therefore the validity of Buddhist practice(s) such as non-self (e.g., sacrificing individual’s own karmic merit in the case of bribing to devote karmic merit to the community) is measured by efficacy, not by its conformity to moral principles or epistemology (Della Santina, 1986, p. 69).

Particularly, skilful means assisted our participants to realize their attachment to the practice of non-self.

There is no ideal self or non-self, but I often forget that in my everyday working context, especially with the stress and expectations that I have to deal with at the workplace. So for me, it is a skilful continuous process of trying to de-attach from my illusions of having a ‘self’/’non-self’. But it is crucial not to be attached to that process as well because you practice differently at different times, under different pressures and organizational contexts. That is for me the resemblance of skilful means and wisdom-enacted practice in Buddhism. Not everything operates as a formula so it is important that you are being skilful. (B52/VN28)

The above participant reaffirmed that the mechanism of skilful means enabled him to critically evaluate the way he practiced non-self to avoid over-attachment, which was shared by a number of participants who considered skilful means as method to apply Buddhist teachings wisely:

The Lotus Sūtra (*Diệu pháp Liên hoa kinh*) (*Saddharma Puṇḍarīka Sūtra)* helped me to realize that all Buddhist practices are skill in means, there is no one best way to practice them. For me, non-self is not about having to reject the ‘self’ to learn to be compassionate. It is a means to transcend all forms of attachment, even to the practice itself when it is no longer relevant or useful. My experience is that it is not always helpful to be compassionate all the time as a leader because others take advantage of it. Trying too hard to practice is also attachment. For me, that is wisdom. (B21/VN13)

As practitioners from the Mahayana tradition, participants in our study highlighted the importance of *The Lotus Sūtra* as a critical tool to debate over Buddhist practices including non-self. The practice and doctrine of Skillful Means reflect the non-foundational nature of Buddhist practices to warn against becoming too attached to the Buddha’s teaching without critical evaluation (Schroeder, 2011).

However, some participants commented that they have not been able to master non-self skilfully and context-sensitively as applying non-self as a Skilful Means requires continuous effort in practices, and may even failure along the journey. Many participants shared that they appreciated the journey as a learning experience of the Buddhist practice, following the guide from the Avataṃsaka Sūtra:

Non-self is a tricky practice. It is like fishing, you have to know when to tighten or loosen the fishing rod. The joy of fishing is not about catching a fish, but enjoying the learning experience and moments of fishing. Non-self is the same. The more you are attached to master non-self, the more you are likely to fail […] for me, it is a learning curve, a journey, not a destination that matters as the Buddha taught this Bodhisattva path in the Avataṃsaka Sūtra (*Kinh Hoa Nghiêm*) […] (B2/VN2)

#### The Middle Way

Participants also stressed the need to balance the internal/external expectations which influence how they see and do things. To do so, they embraced the notion of the Middle Way. To illustrate the Middle Way, one participant highlighted that:

In Buddhist practice, balancing your own and others’ expectations is very important. It is called the Middle way. Without such a balance, you only end up fulfilling your own imagination of your ‘self’ […] or promoting the ‘self’ that others’ expect to see from you […] in the name of non-self while being reluctant to evaluate others’ intent in their expectations […] What if others expect me to do things that involves bad karmic consequence for myself and others? What if they take advantage of the practice of non-self?. The trouble of extremely rejecting the self or trying to practice non-self at all cost is to become reluctant without the ability to judge the situation. Neither reflects the true understanding of non-self and its ethical orientation to bring benefit for others […]. (B1/VN1)

The above participant stressed that there is a need to avoid falling into extremeness of substantialism and nihilism that overly rely on the existence of the self or fully rejecting the self by being overly attached to the practice of non-self, resulting in reluctance without the ability to respond context-sensitively. Without considering the karmic merit or demerit of extreme approaches, one cannot recognize the moral responsibilities for the consequences of actions that conform to the Buddhist doctrine of karma since karmic consequences need to be considered in evaluating an action, and motives and intentions control the kind of karma we receive from action (Fink, 2013; Keown, 1996). Participants emphasized the need to recognize how their practice promotes the dedication of the karmic merit to the benefit of others, not for the ‘self’ following their Mahayana tradition (Clayton, 2013):

I always try to remind myself about why I am practicing Buddhism, what does non-self really mean to my practice and to my life and work. To avoid diverting myself from being a reluctant or arrogant colleague, I keep reminding myself that to transform myself, I need to learn to be compassionate, starting with learning to appreciate the meaning behind doing good things, helping out colleagues and happily dedicating karmic merit and good deeds to others without being instrumental. That is the theory, but it takes years to master. (B40/VN20)

Participants recognized that there is no easy way or short cut to master non-self as it is a transformative practice coming though the performance of virtuous deeds and experiential consequences to enact moral self-transformation (Keown, 1996). Practicing non-self should not be extreme to fall into the state of ‘indulgence’ (Schroeder, 2004) in a form of instrumentalization.

The Middle Way is also a crucial practice to balance/moderate the level of expectations (both individual and collective/organisational), which is crucial to the practice of non-self since expectations are largely associated with strong desires which lead to suffering.

Balancing expectations are crucial. Too much of them can lead to greed and desires leading to suffering, but having none shows reluctance that does not demonstrate personal effort and willingness to accept failure, which is part of the practice. For my leadership, it is extremely important to learn from my mistakes and from my people. It is not fair to abandon them, which I believe, I still do, which is my shortcomings. (B20/VN12)

All participants in our study practiced the Middle Way as a method to balance internal attitudes and external action that takes into consideration an equanimity towards excessive attachment. This mechanism highlights how emptiness as well as all empty phenomena are empty in a sense that all phenomena and emptiness in itself are conventionally existent but ultimately empty (Murti, 1973). In other words, reification of the self with failure to note the impermanent nature of phenomena and being reluctant derived from nihilism in failing to recognize the dependent arising nature of phenomena can all lead to suffering. All phenomena including the notion of the self has a relational and processual nature rather than being permanent (Chinn, 2006).

## Discussion

**Fig. 1:**
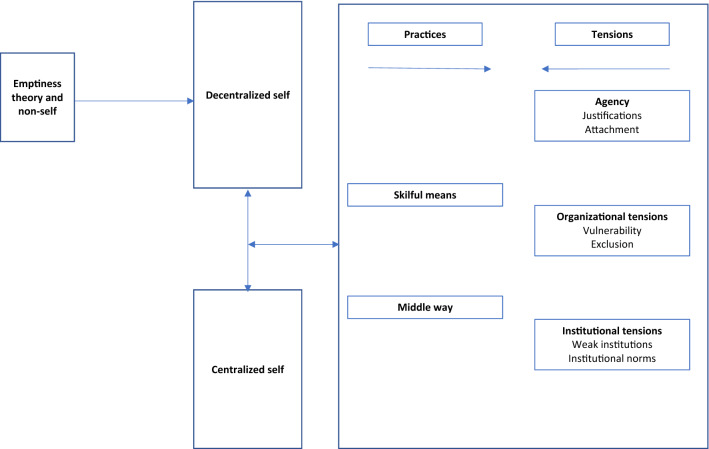
The process of self-decentralization

In this paper, we have unpacked how self-decentralization has emerged as a strategy for moral reasoning enacted through the practices of *Skilful Means* and *the Middle Way*, and which results in moral reasoning that gives primacy to social outcomes. However, we also find that the work organization creates significant contextual tensions which lead to pressures to ‘re-center’ the self as an ‘enlightened’ self. These contextual pressures lead to changes in moral reasoning to more individualistic concerns. Self-decentralization as a moral reasoning strategy, challenged by an entwinement of agency and contextual adjustments, is shown in Fig. 1.

The study makes a number of contributions to the business ethics literature. First, the study introduces a fine-grained conceptualization of the under-developed notion of self-decentralization (Vu, 2021b). We have shown that for Buddhist leader-practitioners self-identity-construction is immersed and embedded in the practice of self-decentralization, which constitutes a process—a spiritual journey—towards the cultivation of a *decentralized self,* where the ego is emptied of the self, and the self becomes “empty of specific materiality” (Schuyler, 2012, p. 6). Interpreting *self-decentralization* based on the Heart Sūtra (*Bát Nhã Tâm Kinh*), Buddhist leader-practitioners described it as a process to eliminate human suffering from excessive attachments or fulfilment of ego. In particular, it is interpreted as letting go of *ego-centric desires* that strive for recognition, *ego-serving knowledge* that results in presumptions and justifications that prevent true wisdom, *ego-serving expectations* that reflect levels of attachment and suffering if expectations are not met, and illusions of having a self that govern behavior towards the fulfillment of self-hood. Further, this interpretation of self-identity is distinct from ideas of an integrated and whole self as frequently described in the SAW literature (Dehler & Welsh, 1994; Lips-Wiersma, 2003; Neal, 1999). In contrast, our participants rejected these kinds of interpretations and affirmed that any desire to attain a whole or integrated self can reflect ego-centricity which is interpreted as a departure from Buddhist teaching.

Our second contribution highlights two mechanisms of self-decentralisation that regulate moral reasoning: *Skillful Means* and the *Middle Way.* As interpreted by participants, *Skillful Means* emphasizes context-sensitivity based on non-attachment, and individuals constantly deconstruct their context reflexively to identity the ‘right’ skillful means needed in a particular context. For instance, our participants highlighted how skillful means helped them to navigate moral reasoning in the practice of non-self when it comes to bribery. Bribery can become a useful skilful means when it can generate positive collective karmic merit, justified by good deeds in intent and motive. However, bribery will no longer be a skilful means if it does not associate with positive motives or for the benefit of social outcomes. Likewise, even non-self and self-decentralization as a moral reasoning strategy have to be put into context. There needs to be a skilful awareness to identify when over-attachment to self-decentralisation can trigger the return to self-centralisation (Vu, 2021b) that can lead individuals to engage in self-serving moral conclusions (Kunda, 1990). With Skilful Means, individual responsibility is put into context by evaluating the karmic merit and benefit to the community rather than organizational expectations alone. The mechanism of skillful means enables practitioners to recognize the contextual and processual dimensions of self-decentralization in interpreting moral actions and intentions in the interplay between agency and context to avoid negative emotions affecting moral judgments (Cowan & Yazdanparast, 2019). In other words, skilful means enacts practitioners’ awareness in avoiding over-attachment to any spiritual or Buddhist practice and acknowledge context-sensitivity (e.g., Shin et al., 2021; Vu & Burton, 2021) since Buddhist practices are not passive but need to be seen in context: “If you cling to it, if you fondle it, if you treasure it, if you are attached to it, then you do not understand that the teaching is similar to a raft, which is for crossing over, and not for getting hold of” (Conze, 1954, p. 223).

Participants also embraced *the Middle Way* in helping them to balance the internal expectations of pursuing a decentralized-self and the external expectations of maintaining a fixed and stable, but illusionary self in an organizational context. For instance, the Middle Way was explained by participants as a way to moderate their own expectations to move away from negative motives and personal desires and at the same time help participants to avoid over-attachment to the need to fulfil collective/organisational expectations at the expense of individual moral responsibility. This approach therefore contributes and responds to the ongoing scholarly debate on individual moral responsibility versus collective expectations within organisations (Rozuel & Kakabadse, 2010). The Middle Way helps to avoid falling into extremes of self-enlightenment and ego-centric individualism. On the other hand, it enables individuals to avoid fulfilling organizational expectations at all cost. The Middle Way highlights moderation in avoiding extremes of substantialism and nihilism in self-decentralization that can lead to self-enlightenment and ego-centric individualism in pursuing the self at all costs or reluctance in rejecting the self in all circumstances within a non-conceptual insight to eliminate propositional knowledge leading to dualistic interpretations. Without considering the karmic merit and demerit of extreme approaches, one may fail to recognize moral responsibility without the ability to evaluate consequences of actions and intents (Fink, 2013; Keown, 1996). It is important to understand how the self is relational and processual in nature rather than impermanent (Chinn, 2006) and how embracing karmic merit to benefit others, and not the self, can help to navigate self-decentralization.

Self-decentralisation as a strategy for moral reasoning moves beyond moral rationalisation (interpreting the immoral action as less immoral), moral decoupling (dissociates immoral judgments from job-related performance judgement), and the overemphasis on judgements relating to the self-concept (Cowan & Yazdanparast, 2019). The combined mechanisms of Skilful Means and the Middle Way facilitate the idea of a contextual-relational-processual perspective to encourage individuals to cultivate self-decentralization through continuous contextualized practice and learning.

Third, while Buddhist leader-practitioners have a shared interpretation of a decentralized self, its operationalization is complicated in work organization contexts as it is nonetheless challenged by individual agency (through karmic reasoning and re-centering the self) and contextual tensions (organizational and institutional constraints). In our findings, we highlighted two types of agency that affected moral reasoning. First, participants in our study tended to justify their own individuated interpretation of self-decentralization and moral reasoning. For instance, many relied upon karmic reasoning to justify their own moral reasoning, even when the means entailed unethical behavior. The way in which our participants used ‘karma’ as a form of moral reasoning was rather surprising. Rather than utilize karmic reasoning to justify self-serving or individualistic behaviour, karmic reasoning was used to justify unethical means, so long as the ‘ends’ were perceived as collective and social. For example, participants justified acts of bribing officials on the basis that the intention was good and the ends had a positive community outcome. In such circumstances, the means were interpreted as moral, or at least acceptable. Such interpretations reflect the controversial account of karma in terms of samskdras^5^ (samskdra theory of karma) as it shows how morality is reducible to psychology in all respects and how considering intentions as determinative or moral quality can imply that it matters little what we do since wrongdoings are acceptable as long as our attitudes and dispositions are correct (Reichenback, 1990, p. 27).

Our findings also illustrate that Buddhist leader-practitioners experienced strong pressures to ‘re-center’ the self through a temptation to strategize self-identity as an ‘enlightened’ version of self as a kind of leadership brand. For example, participants tended to use karmic reasoning to highlight and promote their own ‘self-enlightenment’ and a kind of ‘holier than thou’ attitude. By framing their moral reasoning around the importance of social outcomes, participants were able to gain a sense of moral superiority in a form of self-enlightenment over those who pursued individualistic ambitions, who they perceived as ‘unenlightened’. In other words, emptying out individual agency is clearly problematic and challenging. Our study suggests that individual agency undermines the process of self-decentralization and runs the risk that individuals construct a fantasized identity with self-justified reasoning to facilitate the ‘enlightened’ version of who they want to be (Brown & Toyoki, 2013).

Finally, our findings help illuminate the contextual constraints of self-decentralization. In our study, individuals negotiated their decentralized self within an organizational context consisting of different values, personalities and expectations (Brown, 2015). Participants in our study highlighted two organizational tensions: *vulnerability* and *exclusion*. First, participants expressed hesitancy to exercise self-decentralization due to the fear of being vulnerable and possibly being taken advantage of by others in the work organization. Self-decentralization opens up vulnerability and can lead to withdrawal and disengagement from group dynamics. Thus, exercising self-decentralization in a leadership role can create vulnerability and recourse to defensive identity work as vulnerability is associated by our participants with exposure to harm (Knights & Clarke, 2014), and being an easy target (Thomas & Linstead, 2002). Second, Buddhist leader-practitioners experienced a feeling of perceived workplace exclusion. In work organizations where the leadership team does not universally share a spiritual tradition, our participants often remarked that their attempts at self-decentralization sat uncomfortably with peers. In particular, the Buddhist practice of karmic reasoning often conflicted with the individualistic or economic motives of the work organization. As a result, during managerial processes such as decision-making our participants often experienced being ignored or side-lined, leading to a felt-sense of perceived exclusion. This finding departs from much of the literature that has located spirituality as a positive factor for inclusion (Pfundmair et al., 2015; Pio & Syed, 2018). In contrast, our findings show that self-decentralization in complex organizational contexts can be a painful and even counterproductive experience in a sense that participants’ moral responsibility to deliver social outcomes is negotiated alongside organisational norms and expectations and can sit uncomfortably alongside contrasting moral standards. Such experiences reflect the journey of the Bodhisattva path in learning to interpret phenomena at the ultimate level of truth through understanding and being exposed to experiences of emptiness as highlighted by our participants through their understanding of how the Buddha taught emptiness in the Avataṃsaka and Laṅkāvatāra Sūtra. The struggles that participants faced in the process of self-decentralization and moral reasoning reflect the stages (ten bhūmis)^6^ that a Bodhisattva undergoes for the cultivation of moral and spiritual perfections (Conze, 1968). As highlighted by the participants, self-decentralization was part of their Buddhist journey that many were still trying to master. They acknowledged that the journey is not without painful experiences and lessons since it is not until the seventh bhūmi (stage) that a practitioner attains wisdom through earlier stages of cultivating morality and accumulating knowledge and intellectual development gradually (Yu, 1974). Therefore, the struggles of self-decentralization in moral reasoning would likely to dissolve when practitioners are no longer disrupted by discerning, non-discerning or dualistic perceptions; and acknowledge the absence of an absolute self-existent substance or a substratum in all phenomena in the later stages (eighth, ninth, tenth bhūmis) of the Bodhisattva journey.

In our case analysis, we also find that institutional structures normalized particular forms of individual behaviour. This normalization process acts as a contextual force on Buddhist leader-practitioners to continually renegotiate the normative tensions between their spirituality and the culturally-embedded behaviours that the institutional context treats as normalized. Vietnam reflects a transitional context with moral ambiguity (Zheng et al., 2014) and characterized by a lack of trust in enforcement by institutions and society (Vu & Tran, 2021) and weak law and regulation (Hoskisson et al., 2000). In this institutional context, participants remarked that they saw a pivotal role for spirituality to act as an informal institution to regulate workplace ethics (Helme & Levitsky, 2004; Neubert, et al., 2017). The perspective that spiritual practice is affected by context (e.g., Assouad & Parboteeah, 2018; Neubert, et al., 2017; Parboteeah, et al., 2009), and spirituality (and religion) are institutions that guide individual behaviour and social norms (e.g., Assouad & Parboteeah, 2018) has been previously noted. However, in our study participants recognized the many challenges of pursuing self-decentralization in a context characterized by weak institutions. For instance, as we noted, many participants drew upon karmic reasoning as a form of moral reasoning to respond to weak institutional frameworks by articulating that unethical means—for example, the act of bribing officials—had to be weighed against the positive outcomes that the means may bring to the social community. While the normative aspect of Vietnam’s institutional context reflected values and ethics often inconsistent with the normative guidelines of Buddhist teaching, the use of karmic reasoning to resolve the problem of incompatible forms of moral reasoning contributes further to our understanding of structures of moral reasoning and illuminates further the potential relationship between spirituality and (un)ethical behaviour.

The complex organisational and institutional contexts in this study highlights how the notion of moral reasoning is heterogeneous across social and cultural contexts (Haidt, 2001) and the need to deconstruct the complex agentic and contextual factors impacting on moral reasoning is clear. A context of weak institutions, for example, shows how culture influences moral reasoning (e.g., Tsui & Windsor, 2001) and can generate tensions relating to an individuals’ moral standards (Cowan & Yazdanparast, 2019).

In this paper, we conceptualized self-decentralization from a Buddhist perspective as a contextual-relational-processual approach. Such an approach contributes an Asian lens to conceptions of the self in moral reasoning. Self-decentralization further extends the *relational view* about the nature of the self in the concept of *Oneness* (primary moral aspects are embedded in the relationship between the self and ‘other’) (Ivanhoe, 2017; Ivanhoe et al., 2018)*.* Besides the Confucian and the Dao perspectives on Oneness (Ivanhoe, 2017), we introduce the notion of self-decentralization from the Buddhist perspective with mechanisms to decenter the self and to reject ego-serving pursuits to recognize the importance of the interests of others. By recognizing the ‘empty nature of the self’, self-decentralization stresses the need to take into consideration the dependent-arising nature of all phenomena and collective social outcomes in moral reasoning through karmic reasoning. It further extends Vokey’s (2001) non-foundational justification view by showing how adopting the Middle Way and Skillful Means in self-decentralization can enable ‘reflective equilibrium’^7^ by moderating desires/expectations and *making sense of the given context of enquiry and practice* in moral reasoning. The ‘empty’ nature of self-decentralization also highlights the *processual nature* in reflecting equilibrium as non-foundational and non-circular by facilitating the rejection of assumptions and commitment in a particular context as they are subject to revision or rejection as the process unfolds (Vokey, 2001, p. 94). Though there are tensions and struggles identified in self-decentralization, those experiences are necessary efforts along the process to uncover the limitations of prior perceptions of any tradition of enquiry. Such experiences are salient in the Mahayana metaethics and practice as it highlights the journey of self-correcting in seeking reflective equilibrium given the fact that no spiritual journey even within the same tradition is the same (Vokey, 2001). The Lotus Sūtra serves as a critical tool through the practice of skillful means to question Buddhist practices as a moral tradition (e.g., non-self) as norms and fundamental intentions are subject to be revised or replaced to maintain reflective equilibrium. Furthermore, the heart of skillful means is compassion (Schroeder, 2004), similar to how Vokey (2001, p. 232) referred to the ‘reasons of the heart’ as a Mahayana view of wisdom and a non-dualistic apprehension of intrinsic moral value to show what is good is relative to human interests and what is ‘good in itself’ vanishes due to an ‘either-or’ perspective that does not arise in emptiness. In other words, individuals should perceive intrinsic goodness in its empty form in moral reasoning rather than judging goodness with reference to human interests.

Our study also has some practical implications for ethical leadership beyond the Buddhist context as the need to enhance leaders’ moral competencies remain as pressing as ever. We recognize the challenges of transferring philosophical ideas and practices from one tradition to another (MacIntyre, 1964), and the risks of fragmentation and distortion of Buddhist spiritual practices when taken up by organizations to support instrumental ends (Vu, et al., 2018). For instance, when the practice of mindfulness was utilized by predominantly Christian leaders, mindfulness was found to have limited impact upon moral reasoning (Small & Lew, 2021) suggesting that a nuanced understanding of its liberative and social ambition is essential to leverage its potential in secular contexts. This presents a conundrum. Nonetheless, if moral reasoning through the doctrine of non-self represents a stage of social cooperation where personal self-interest is low (Ryan, 2001), then embracing a conception of non-self is an intriguing proposition for ethical leaders. If ethical leaders can embrace a journey towards non-self through a practice of universal compassion towards others, then there is a possibility that leaders can maintain consistency with moral principles when making moral judgements. For example, relying upon principles of compassion and other-orientation may encourage leaders to frame decision-making and leadership in a way that reflects the collective well-being of colleagues and stakeholders. In turn, the practice of these kinds of prosocial behaviour has the potential to permeate an organization and its culture (Burton & Vu, 2021). We caution, however, that embracing Buddhist principles/practices, such as non-self, is nonetheless challenging, and the tensions associated with non-Buddhist leaders’ utilizing non-self as a process for self-transformation remain ripe for further exploration in a number of different organizational, sectoral, and cultural contexts.

Furthermore, most contemporary Western theories of the self implies a form of hyper-individualistic conception of the self (Berger, 1983; O’Neill, 1993; Rawls, 1999) that is concerned with self-actualization and self-fulfilment embedded in ideas of moral cosmopolitanism, idealization, abstraction, and acontextuality that maximize own interests without moral obligations to make society better (e.g., Callicott, 1989; Ivanhoe et al., 2018; Miller, 2012). This lens is problematic, particularly in the context of, for example, the Covid-19 pandemic since an individuals’ self-concept of ‘I’ or ‘we’ dictates the prioritization of self and immediate kin as opposed to the wider community, and which seems to have led to more cases and mortalities in cultures with hyper-individualistic orientations due to a unwillingness to adhere to epidemic prevention measures (Maaravi et al., 2021). Therefore, it is important for both individuals and organizations to recognize how the ‘self’ is relational and processual in nature rather than situating the self as definite and enduring, which can be a source of suffering (Ivanhoe et al., 2018). Organizational practices should aim at promoting a culture and psychological contracts that extend beyond an emphasis of a desired self that can colonize organizational members’ identities and humanity in organizations (Johnsen & Gudmand-Høyer, 2010). Promoting the self as de-centred can enhance organizational capacity for compassion (Madden et al., 2012) since individuals can modify their roles, behaviours, group norms from self-interest to spread compassion through the organization via their interaction with other stakeholders (Grant & Patil, 2012; Rynes, et al., 2012).

## Conclusions and Future Research

Our study has analyzed the Buddhist theory of emptiness and provides a fine-grained articulation of the practice of self-decentralization involving Skilful means and the Middle way as a strategy for moral reasoning. We have shown how self-decentralization is utilized as a strategy for moral reasoning that foregrounds collective social outcomes as a moral frame. However, we have also revealed the dynamic tensions that influence how individuals connect the notion of the decentralized self to moral reasoning and negotiate between perceived moral responsibilities and organisational and institutional expectations (Reed et al., 2007; Rozuel & Kakabadse, 2010; Treviño et al., 2006). We have drawn attention to tensions to do with agency using karmic reasoning and re-centering the self, and how contextual challenges influence the negotiation between self-decentralization and self-re-centering. Our study contributes an alternative perspective on ethical business beyond the argument that business is embedded in society (Demuijnck & Fasterling, 2016) but is essentially a part of the larger society through the idea of Oneness (Ivanhoe, 2017), grounded in a humanistic approach with a shared connection with others. In other words, business ethics today needs to be understood beyond rationalism and individualism, taking into consideration diversity and cultural settings within the field with an anthropocentric perspective on corporate citizenship and stakeholder theory, such as the Asian idea of Oneness (Ivanhoe, 2017, Ivanhoe et al., 2018) embedded through the notion of self-decentralization.

Our work offers a window into the challenges and tensions experienced by our participants in following the Buddhist path. We found their experiences of vulnerability and exclusion, and the challenges involved in operationalizing self-decentralization as a strategy for moral reasoning both intriguing and unsettling. Furthermore, we hope that our work demonstrates the challenges of self-decentralization, but at the same time, how the path towards attaining such a state could lead to forms of moral reasoning that challenge self-idealization and organizational norms. Our conceptualization of self-decentralization also challenges the dominant narrative of a ‘whole self’ identity in the SAW literature, and we hope that further research will expand upon our findings.

We identify a number of possible research pathways. First, our finding that spiritual practice can lead to vulnerability and exclusion in leadership opens up a pathway for future research that examines spirituality and vulnerability. Further, Corlett et al. (2019) have argued that vulnerability could be reconceptualised as a strength and become the norm in managerial identity and learning. How spirituality can help recast vulnerability as strength would be welcome. Second, the extent to which spirituality interacts with different institutional and cultural contexts requires further scholarship. For example, Zheng et al (2019) found strong evidence that individuals with strong religiosity are more likely to engage in entrepreneurship in well-developed markets, and less so in markets characterized by corruption. Their findings suggest that entrepreneurs seek to avoid a disharmony between religious beliefs and the institutional context. Pointing to new pathways, Neubert et al. (2017) found that spiritual capital fills institutional voids and may be an important resource in transitional economies. We hope further research will examine the interplay between spirituality and institutional contexts. We encourage future studies to examine the notion of spiritually-enacted forms of moral reasoning from different spiritual and religious traditions to explore mechanisms that regulate moral reasoning based upon philosophy, theology or other forms of belief structure.

## Footnotes

1 Reflects a view that there is no ultimate reality, there is no ultimately true reductive base for an analysis of persons (Finnigan, 2020).

2 Reflects a form of metaphysical idealism whereby personal identity is analysed as mere reifications of the structural features of conscious experience (Finnigan, 2017, 2018).

3 When characterizing a thing, we give it a conventional existence with verbal designation but it retains its fundamental emptiness (Garfield, 1995).

4 Mahayana Buddhism (the ‘Great Vehicle’) reflects a philosophical argument against dualistic assumptions, particularly the conventional belief that subjects and objects have inherent existence to demonstrate non-conceptual insight to transcend limitations of propositional knowledge. The most distinguishing characteristics of Mahayana Buddhism is the notion of *bodhisattva*, someone with a spiritual realization who works tireless for the benefit of others (Vokey, 2001).

5 Invisible dispositions or tendencies to act, think and interpret experiences in ways to designate the transformative effect that moral action has upon the character of the agent (Keown, 1996).

6 The ten stages of the Bodhisattva career is first presented in the Sūtras of the Perfect Wisdom (Conze, 1968): (1) *pramuditā*—joyous, (2) *vimalā*—pure, (3) *prabhākarī*—light-giving, (4) *arcit-gi*ī—radiant, (5*) sudurjayā*—hard to conquer, (6) *abhimukhī*—face-to-face, (7) *dūrae-to-*—far-going, (8) *acalā*—immovable, (9*) sādhumatī*—of good thought, (10) *dharmameghā*—clouds of Dharma.

7 Refers to dialectical interactions within and among all levels and forms of sense-making activity with a goal to achieve the most overall satisfactory set of agreements in a given context of enquiry and practice, where satisfactory means coherent, mutually supporting, successful, and defensible (Vokey, 2001, p. 92).
